# Structure, morphology, and photoluminescence of porous Si nanowires: effect of different chemical treatments

**DOI:** 10.1186/1556-276X-8-383

**Published:** 2013-09-11

**Authors:** Ioannis Leontis, Andreas Othonos, Androula G Nassiopoulou

**Affiliations:** 1 NCSR Demokritos, IMEL, Terma Patriarchou Grigoriou, Aghia Paraskevi, Athens 153 10, Greece; 2 Research Center of Ultrafast Science, Department of Physics, University of Cyprus, P.O. Box 20537, Nicosia 1678, Cyprus

**Keywords:** Si nanowires, Metal-assisted chemical etching, Porous silicon nanowires, Photoluminescence, Structure, Morphology

## Abstract

The structure and light-emitting properties of Si nanowires (SiNWs) fabricated by a single-step metal-assisted chemical etching (MACE) process on highly boron-doped Si were investigated after different chemical treatments. The Si nanowires that result from the etching of a highly doped p-type Si wafer by MACE are fully porous, and as a result, they show intense photoluminescence (PL) at room temperature, the characteristics of which depend on the surface passivation of the Si nanocrystals composing the nanowires. SiNWs with a hydrogen-terminated nanostructured surface resulting from a chemical treatment with a hydrofluoric acid (HF) solution show red PL, the maximum of which is blueshifted when the samples are further chemically oxidized in a piranha solution. This blueshift of PL is attributed to localized states at the Si/SiO_2_ interface at the shell of Si nanocrystals composing the porous SiNWs, which induce an important pinning of the electronic bandgap of the Si material and are involved in the recombination mechanism. After a sequence of HF/piranha/HF treatment, the SiNWs are almost fully dissolved in the chemical solution, which is indicative of their fully porous structure, verified also by transmission electron microscopy investigations. It was also found that a continuous porous Si layer is formed underneath the SiNWs during the MACE process, the thickness of which increases with the increase of etching time. This supports the idea that porous Si formation precedes nanowire formation. The origin of this effect is the increased etching rate at sites with high dopant concentration in the highly doped Si material.

## Background

Si nanowires (SiNWs) are interesting building blocks of different nanoelectronic devices [1-3], solar cells [4,5], and sensors [6]. There are different techniques to fabricate vertical SiNWs on a silicon wafer, which include bottom-up methods using catalysts to initiate nanowire growth [7] and top-down methods using either advanced lithographic techniques, combined with anisotropic etching [8], or chemical etching catalyzed by metals (metal-assisted chemical etching (MACE) method) [9,10]. This last method is a simple low-cost method that permits to obtain vertical Si nanowires on the Si wafer with length that can exceed several tens of micrometers. It can be implemented either in a single step in a metal solution (usually AgNO_3_), involving nucleation of a metal catalyst from the solution on the Si surface, followed by metal-assisted anisotropic chemical etching in the same solution [11], or in two steps, where the metal catalyst is first deposited on the Si surface and the subsequent etching occurs in a second hydrofluoric acid (HF) solution, usually containing H_2_O_2_ as an oxidant agent [12]. The obtained SiNWs are vertically oriented, following the crystallographic orientation of the Si wafer. Depending on the resistivity and type of the parent Si wafer and the fabrication conditions used, the structure and morphology of the SiNWs are different. The SiNWs that result from the etching of highly doped Si wafers show a porous structure [11-19]; however, the question if the nanowires are fully porous or they contain a Si core and a porous Si shell is still pending. The photoluminescence (PL) from porous SiNWs by MACE was investigated in a number of recent papers [13-19].

In this work, we investigated the structure, morphology, and photoluminescence from SiNWs fabricated by a single-step MACE process on highly doped p-type (100) Si wafers with a resistivity of approximately 0.005 Ω·cm and the effect of different surface chemical treatments on the above. We used scanning and transmission electron microscopy to demonstrate that the obtained nanowires were fully porous, and this result was further supported by the fact that they were fully dissolved in an HF solution after successive HF and piranha treatments. We also demonstrated that a porous Si layer is formed on the Si wafer underneath the SiNWs, the thickness of which increases with the increase of the etching time. The chemical composition of the surface of the Si nanostructures composing the porous Si nanowires was investigated after each chemical treatment and correlated with their photoluminescence properties.

## Methods

SiNWs were fabricated on highly doped (100) p-type Si wafers (resistivity of approximately 0.005 Ω·cm) using a single-step MACE process. The samples were cleaned with acetone and propanol, dried in nitrogen blow, and immersed into the etching chemical aqueous solution that contained 4.8 M HF and 0.02 M AgNO_3_. The temperature of the solution was 30°C, and the immersion time was either 20 or 60 min. After etching, the samples were dipped into 50% HNO_3_ to completely dissolve the Ag dendrites and any other Ag residues that were formed on the SiNW surface [20]. The as-formed SiNWs were then subjected to different successive chemical treatments, including a dip in 5% aqueous HF solution at room temperature for 10 min and piranha cleaning in 1:1 *v*/*v* H_2_O_2_/H_2_SO_4_ solution for 20 min. Piranha cleaning is an oxidizing process, while the HF chemical solution removes any native or chemical oxide from the Si surface.

The SiNW morphology was characterized by field-emission scanning electron microscopy (SEM) (JEOL JSM-7401F, JEOL Ltd., Akishima, Tokyo, Japan) and transmission electron microscopy (TEM). Their surface chemical composition was characterized by Fourier transform infrared spectroscopy (FTIR). Finally, PL measurements were carried out using a HeCd laser excitation at *λ* = 325 nm. The PL signal was dispersed by a single-grating monochromator and detected by a photomultiplier. Time-resolved PL measurements were performed by pumping to steady state, mechanically switching off the pump beam, and detecting at a fixed wavelength the PL intensity as a function of time.

## Results

### Structure and morphology

**Figure 1:**
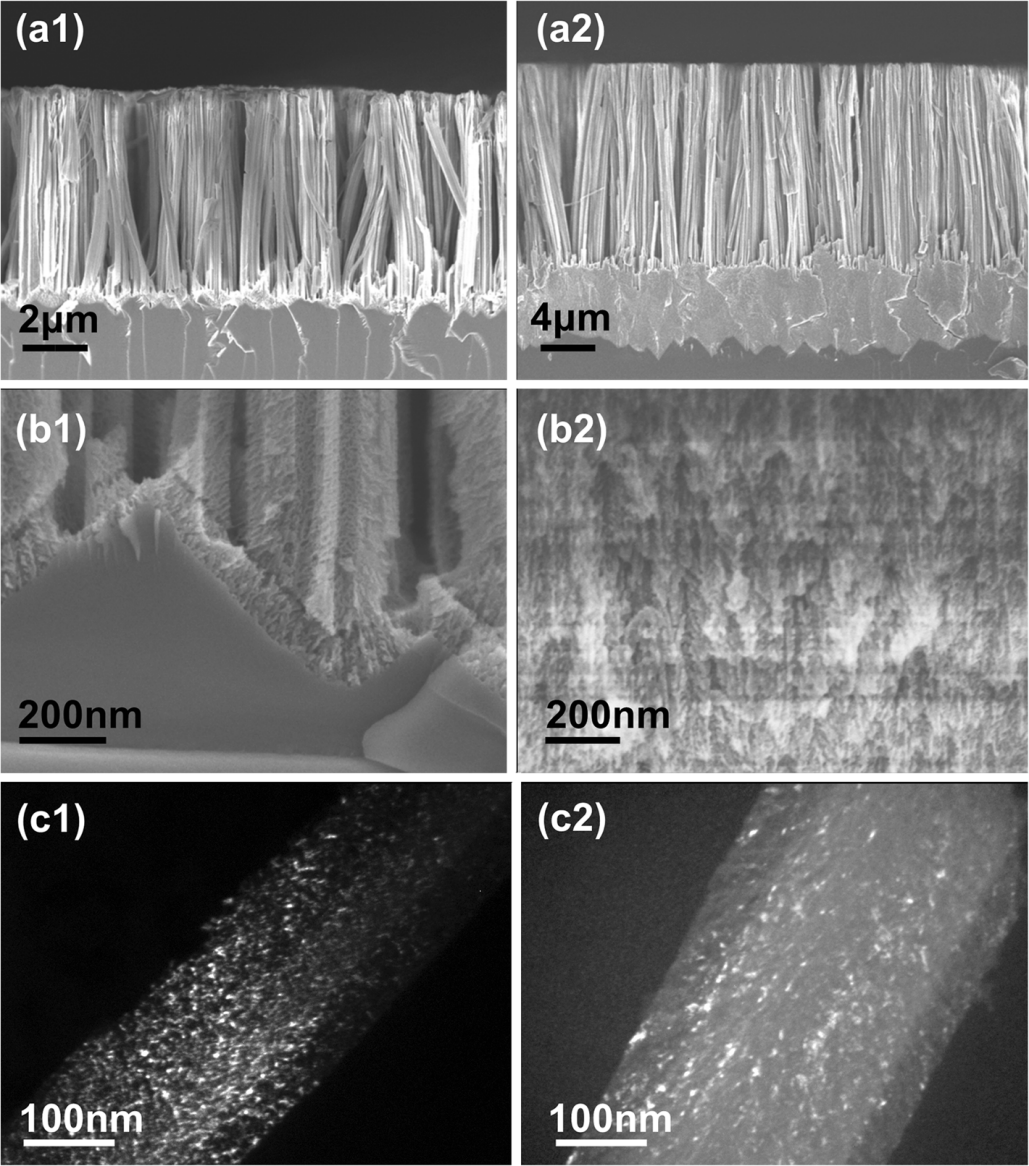
**SEM and TEM micrographs from SiNWs on highly boron-doped Si.** Cross-sectional SEM and TEM micrographs of long porous SiNWs on p^+^ Si (resistivity 0.005 Ω·cm) etched for 20 min **(a1**, **b1**, and **c1)** and 60 min **(a2**, **b2**, and **c2)**, respectively. Micrographs **(a1)** and **(a2)** are SEM images of the nanowires at low magnification and illustrate the existence of a porous Si layer at the interface between the nanowires and the Si substrate. This layer is thicker in the case of the longer etching time, and its structure is porous as it clearly appears in the SEM images **(b1)** and **(b2)**, obtained at higher magnification. On the other hand this layer is thinner in the case of the 20-min etching time, as illustrated in **(b1)**. Micrographs **(c1)** and **(c2)** are dark-field TEM images of the same nanowires etched for 20 min **(c1)** and 60 min **(c2)**, respectively.

Examples of SEM and TEM images of SiNWs resulting from long etching times (20 and 60 min) of p^+^ Si (resistivity 0.005 Ω·cm) are depicted in Figure 1. Micrographs (a1) to (c1) correspond to the 20-min immersion time, while micrographs (a2) to (c2) correspond to the 60-min immersion time. Dense and uniformly distributed SiNWs were formed on the whole Si surface, contrary to what was reported in [11], where the authors mention that only approximately 40% of their Si surface was covered by the SiNWs. The SiNW length was about 6 μm for the 20-min etching time (a1) and about 18 μm for the 60-min etching time (a2). Their average lateral size was approximately 100 nm in both cases, their cross-sectional shape being ‘celery stick-like.’ This size depends mainly on the concentration of Ag ions in the solution. The distance between the nanowires varied between few nanometers and few tens of nanometers. The micrographs (b1) and (b2) show the interface between the nanowires and the Si surface underneath them. It is clearly deduced from these micrographs that this interface is not sharp but shows an important undulation at the SiNW base. In addition, a porous Si film is formed at the SiNW base, whose thickness increases with the increase of the etching time. The thickness of this film was about 0.1 μm for the sample etched for 20 min and about 5 μm for the sample etched for 60 min. The pore size in this film was less than 20 nm (mesoporous film). In our opinion, the formation of this film is at the origin of the mesoporous structure of the SiNWs from p^+^ Si wafers. The presence of such a porous Si film at the interface between the SiNWs and the Si substrate was also reported recently by To et al. [19] for SiNWs formed on n^+^ Si wafers. This will be discussed in more detail below.

Dark-field TEM images of single SiNWs obtained as above are depicted in Figure 1 (c1) for the 20-min etching time and Figure 1 (c2) for the 60-min etching time. These images clearly show that the SiNWs are fully porous, without any continuous Si nanowire core, but composed of small Si nanocrystals (NCs) interconnected in a Si skeleton in their whole volume, as in the case of the porous Si films. The size of these Si NCs ranged from 1 to 20 nm. Additional evidence that the SiNWs were fully porous will be given below by considering the effect of different chemical treatments on their structure and morphology.

**Figure 2:**
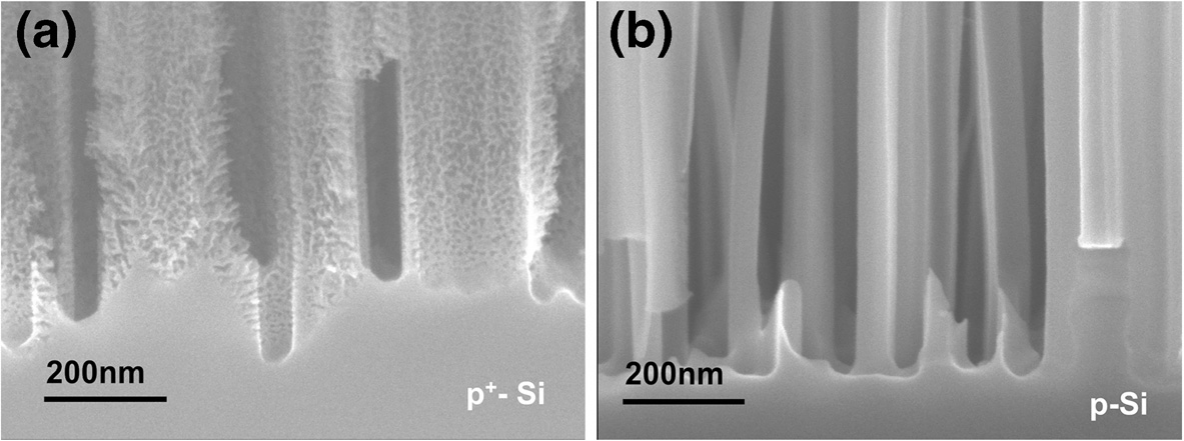
**SEM micrographs of porous versus nonporous SiNWs.** Cross-sectional SEM images of **(a)** porous Si NWs versus **(b)** nonporous SiNWs. Both are etched for 6 min. In both cases, the length of the SiNWs is small (about 1 μm). The porous SiNWs are fabricated on p^+^-type Si (resistivity 005 Ω·cm), while the nonporous SiNWs are fabricated on p-type Si (resistivity 1 to 10 Ω·cm). Due to their small length, there is no clear evidence of the presence of an interfacial porous layer between the SiNWs and the Si substrate.

Short SiNWs on p^+^ Si formed at shorter etching times are also porous; however, no porous layer at the interface of the nanowires with the Si substrate is discerned. Figure 2 illustrates the above for approximately 1-μm-long nanowires (Figure 2a), compared to the nonporous SiNWs obtained on p-type (resistivity 1 to 10 Ω·cm) Si (Figure 2b).

### Effect of different chemical treatments

**Figure 3:**
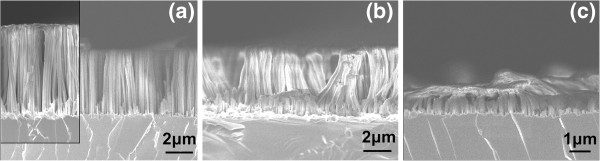
**SEM micrographs of the SiNWs after different chemical treatments.** Cross-sectional SEM images of SiNWs formed at 20-min etching time, after different chemical treatments. In **(a)** the nanowires after an HF dip are depicted. The as-formed nanowires are depicted in the micrograph in the inset of **(a)**. The nanowires after successive. In **(b)** and **(c)** the nanowires after successive HF/piranha **(b)** and HF/piranha/HF **(c)** treatments are shown. After the last treatment, the nanowires were almost fully destroyed.

As-formed SiNWs were subjected to successive chemical treatments in diluted HF and piranha chemical cleaning. Immersion in HF removes the silicon oxide from the SiNW surface, while piranha cleaning is an oxidizing process. Figure 3 shows representative SEM images of SiNWs formed at the 20-min etching time and subsequently subjected to an HF/piranha treatment and a cycle of HF/piranha/HF treatment. The as-formed nanowires are depicted in the inset of Figure 3a. Figure 3a shows the nanowires after an HF dip, and Figure 3b, c shows the nanowires after successive HF/piranha and HF/piranha/HF chemical treatments. From these images, it is deduced that after the first HF/piranha treatment, the length of the SiNWs was reduced from about 6 to about 5 μm, while with the additional HF dip, the SiNWs almost disappeared and only the thicker nanowire base, approximately 1 μm in height, remained.

The above behavior is understood as follows: With the HF/piranha treatment, the SiNWs were partly oxidized. The formed oxide covers all the internal surface of the porous nanowires and leads to expansion of the volume of the Si nanostructures composing the SiNW skeleton (Figure 3b). With the additional HF dip, the SiO_2_ layer from the internal porous Si surface is dissolved, leading to full dissolution of the upper length of the nanowires, which is highly porous (Figure 3c). This proves that the whole volume of the SiNWs is fully porous and that there is no single-crystal Si core in the nanowires. This was an open question in the literature [11]. The fact that after the first HF/piranha treatment the length of the SiNWs is only slightly reduced, while after the additional HF dip the NWs almost disappear, except of a short nanowire base, indicates that the SiNW porosity is not homogeneous throughout their length, but it is higher at their top and it gradually decreases from the top to the bottom. In addition, the fact that the above chemical treatment did not dissolve the porous Si layer underneath the SiNWs means that the porosity of this layer is lower than that of the SiNWs’ tops. Consequently, in the as-grown sample, this layer is not expected to have a significant contribution to the PL spectrum.

### Photoluminescence spectra

**Figure 4:**
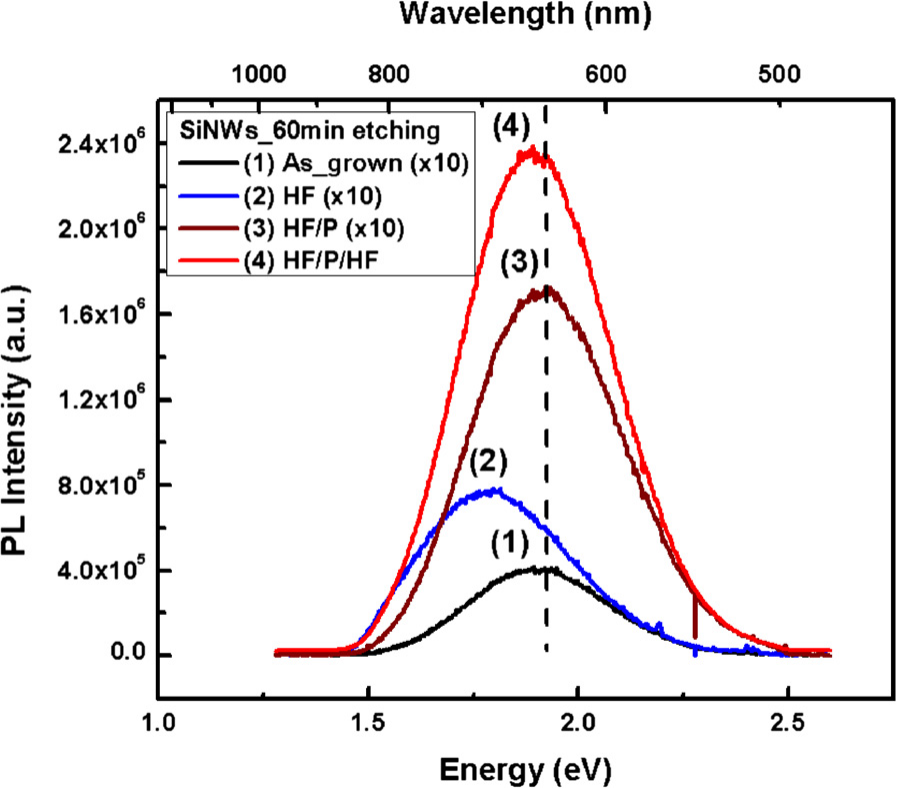
**PL spectra from the as-grown sample etched for 60 min and samples after different chemical treatments.** The spectrum from the as-grown sample is denoted by (1), the sample after an HF dip by (2), after HF/piranha by (3), and after HF/piranha/HF by (4). The vertical dashed line is a guide to the eye.

PL spectra were obtained from the as-formed samples and from samples after different chemical treatments. PL was excited by a HeCd laser line at 325 nm. The results are summarized in Figure 4 for a sample etched for 60 min. The PL peak is broad, with a maximum at approximately 1.9 eV and a full width at half maximum (FWHM) of approximately 380 meV in the case of the as-formed sample. By immersing the as-etched sample into an HF solution, the PL peak was red-shifted from 1.73 to 1.80 eV while the PL FWHM increased from 412 to 447 meV. In addition, the PL intensity increased by a factor of 2. The HF dip was then followed by a piranha treatment that oxidizes the internal Si surface, forming an oxide shell around the nanostructures composing the porous nanowire skeleton. This treatment caused a shift of the PL wavelength to approximately the initial peak energy and the initial FWHM. In addition, the PL intensity was doubled. Finally, after an additional HF treatment, the PL intensity was increased by 50 times, without any significant wavelength shift. These results will be discussed below.

From time-resolved PL measurements, the PL decay time at room temperature was found to be in the 19- to 23-μs range. The decay data were fitted to a stretched exponential decay that in general followed the case of assemblies of Si nanocrystals of different sizes:

$$I\left(t\right)=I_{0}\exp\left(-{\left(t/\tau \right)}^{\beta }\right),$$

where *I* is the luminescence intensity, *τ* is the PL lifetime, and *β* is a dispersion factor which takes values in the range 0 <*β* < 1 [21]. The fitting results for the different samples resulted in a PL decay time in the range of 19 to 23 μs and a constant *β* in the range of 0.85 to 0.95.

The PL results are discussed in detail in the ‘Discussion’ section. The differences in the PL behavior of the different samples can be explained by taking into account that the studied samples constitute very complicated systems of nanowires composed of nanocrystals of different sizes and different surface chemical compositions that, in addition, present different structural defects at their surface. Depending on the chemical treatment, the mean size of the nanocrystals composing the nanowires and their surface chemical composition are different. Moreover, the number and nature of the structural defects change. Both surface composition and structural defects introduce states in the nanocrystal energy bandgap that influence the PL recombination mechanism. In addition, the porous Si layer underneath the SiNWs contributes to the PL signal. The above will be discussed in detail for each sample in the ‘Discussion’ section.

### FTIR analysis

**Figure 5:**
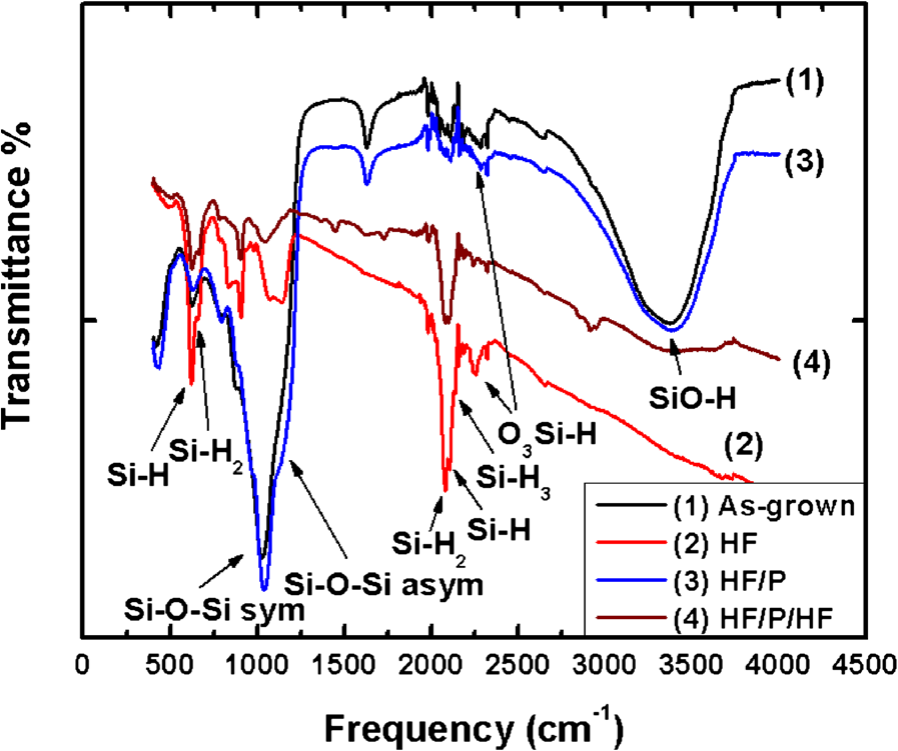
**FTIR transmittance spectra of SiNWs.** The sample was initially etched for 60 min and then subjected to different chemical treatments (as-grown (spectrum 1 - black), after an HF dip (spectrum 2 - red), after HF/piranha (spectrum 3 - blue), and after HF/piranha/HF (spectrum 4 - wine)).

The surface composition of the four different samples was characterized by FTIR transmittance analysis. The results are depicted in Figure 5. The spectra of the as-grown and the piranha-treated samples are similar, showing the characteristic asymmetric stretching signals of the Si-O-Si bridge between 1,000 and 1,300 cm^−1^, with a strong band at 1,080 cm^−1^ and a shoulder at 1,170 cm^−1^[22]. Furthermore, a strong broad signal between 3,000 and 3,650 cm^−1^ is present, attributed to the stretching signal of the SiO-H bond [22]. Finally, the peak at 626 cm^−1^ is in general attributed to the Si-H bond [22]. However, since no other vibrations of the Si-H bond are present, this peak can be attributed to the wagging vibration mode of the OSi-H bond. On the other hand, the FTIR transmittance spectra after the first and the second HF dip (Figure 4, spectra 2 and 4) do not show any significant surface oxide signature, since the surface oxide has been removed by the HF. The characteristic asymmetric stretching signals of the Si-O-Si bridge between 1,000 and 1,300 cm^−1^ and the wagging and stretching points of O_3_Si-H at 847 and 2,258 cm^−1^ are too weak. Instead, the transmittance peaks due to different vibration modes of the SiH_x_ bond (the wagging and stretching vibration modes of Si-H bond at 623 and 2,112 cm^−1^, and the wagging, scissors, and stretch vibration modes of Si-H_2_ bond at 662, 908, and 2,082 cm^−1^) respectively [22] are too strong, corresponding to the hydrogen signature at the SiNW surface. These results are exactly what one could expect from a Si surface after the above chemical treatments.

## Discussion

The structural characterization of samples etched at different etching times provides additional insight to the mechanism of formation of the mesoporous SiNWs on highly boron-doped Si by the single-step MACE process. In principle, MACE involves two successive processes: surface nucleation of metal catalysts (e.g., Ag) and anisotropic Si etching. Si dissolution takes place through oxidation by H_2_O_2_ and oxide dissolution in HF. Metal nucleation occurs preferentially at surface states and sites around the dopants. Since the oxidation donates four electrons, while Ag^+^ ion reduction consumes only one electron, a space charge is formed by the excess electrons on the surface that electrically drives Ag^+^ ions to diffuse toward the nuclei for reduction. Alternate oxidation and nucleation cycles induce sinking of the Ag particles into the Si substrate, resulting in Si etching and SiNW formation. These nanowires are vertical to the Si substrate. The morphology and texturing of the SiNWs depend strongly on the original Si wafer resistivity. SiNWs from resistive Si wafers have in general a smooth surface and a crystalline core without pores. On the other hand, Si wafers with a resistivity of less than approximately 5 mΩ·cm produce mesoporous SiNWs. This was demonstrated for both p-type [11] and n-type Si wafers [12,19]. Since dopants are additional preferential sites for the nucleation of Ag particles, their high density induces porosification of the Si substrate and the formation of a mesoporous layer at the interface between the SiNWs and the crystalline Si substrate. Our experiments showed that the thickness of this porous Si layer increases with the increase of the etching time. It was also deduced from our PL experiments (this is discussed below) that the initial porosity of this layer was lower than that of the SiNWs. Furthermore, the porosity of the SiNWs was gradually increasing from their bottom to their top (different pore and nanocrystal sizes). These observations led us to the conclusion that the formation of the porous Si layer underneath the SiNW arrays precedes the SiNW formation. The SiNWs are thus porous from the beginning, while additional porosification of the nanowires takes place during etching. The higher porosity of the tops of the SiNWs is attributed to the longer time into the etching solution and is responsible for the saturation of the process after a certain time. Indeed, we observed that after the 60-min etching time, it was not possible to further increase the SiNW length. This is attributed to the fact that part of their tops is fully dissolved in the solution when the porosity of this part of the nanowires becomes high enough. From that time on, although the etching process continues on the Si surface, the SiNW length does not increase, since the nanowire tops are progressively dissolved in the solution.

The PL spectra from the mesoporous SiNWs can be understood by taking into account their structure and morphology. As discussed above, the nanowires are composed of assemblies of Si nanocrystals and nanowires interconnected in a Si skeleton, the mean size of these nanocrystals being different along their length. The PL spectra from assemblies of Si nanocrystals are in general broad, and peak position depends strongly on their size distribution and the chemical composition of their surface [21,23-27]. Quantum confinement of the generated carriers is at the origin of the long decay times (in the several micrometer range) [25,27]. The recombination mechanism depends on the structural and chemical composition of the nanocrystal surface. In hydrogen-terminated nanocrystals without important structural defects at their surface, free exciton recombination is in general observed [28,29], while in oxidized nanocrystals, a significant Stokes shift is observed between the absorption and the PL band peak energy [27,30,31], attributed to an important pinning of the nanocrystal energy bandgap due to localized states at the interface of Si NCs with the surrounding SiO_2_ matrix [27,30,32,33]. The same effect can be caused by structural defects at the surface of the nanocrystals. Pump and probe measurements confirmed the above behavior [33].

The differences observed from the different samples investigated in this work can be explained, based on the above, by considering the size distribution of nanocrystals and the state of their surface. In the as-grown samples, a number of very tiny nanocrystals that are light emitting are found at the surface of larger nanocrystals. On the other hand, a lot of structural defects exist that quench luminescence (spectrum 1 in Figure 4). The tiny nanocrystals (slightly oxidized at ambient atmosphere) are removed by the first HF dip. In addition, some of the structural defects that quench PL are also smoothed out. This is why the PL signal from the SiNWs after the first HF dip is red-shifted compared to that obtained from the as-formed nanowires, and its intensity increases (spectrum 2 in Figure 4). The different surface chemistry of the as-formed and HF-treated NWs is confirmed by the FTIR results. In the HF-treated samples, the surface is hydrogen-terminated, while the as-grown sample and the sample after piranha cleaning show mainly Si-O and SiO-H bonds at the surface. The slightly oxidized NWs after piranha cleaning show a blueshift in PL due to a slight shift of the mean nanocrystal size by oxidation (spectrum 3, Figure 4). The increase in intensity is again attributed to a further smoothing of surface structural defects that quench PL. Furthermore, light emission from additional nanocrystals, which were dark before due to their large size and are now smaller after oxidation, contributes to the increased PL intensity.

The large increase in intensity after the last HF dip is attributed to the total change of the structure and morphology of the SiNWs, as illustrated in Figure 2c. This additional HF dip resulted in dissolution of the upper part of the SiNWs. The length of the remaining SiNWs was only the one fourth of their original length. However, even if the SiNW length was significantly smaller, the PL intensity was increased by more than one order of magnitude. To our opinion, PL in this case comes mainly from the mesoporous Si layer underneath the SiNWs. The mean size of NCs in this layer was initially large, while it was reduced by HF/piranha/HF treatments. The peak position is mainly determined by the mean size of the NCs of this layer. Consequently, there is no direct comparison of this spectrum with the three previous spectra.

## Conclusion

The structure, morphology, and light-emitting properties of SiNWs fabricated by a single-step MACE process on p^+^ Si were investigated for samples subjected to different chemical treatments after the SiNW formation. The investigation of the structure and morphology of the nanowires revealed that their whole volume was porous, this being also confirmed by the fact that after successive HF and piranha treatments, almost all the upper part of the vertical nanowires was fully dissolved in the chemical solution, leaving only their less porous nanowire base intact. Hydrogen-passivated SiNWs showed shifted PL spectra compared to the oxidized ones, due to defects at the interface of the Si nanocrystals with the SiO_2_ shell that are involved in the PL recombination mechanism. All the obtained results concerning light emission and structural characteristics of the SiNWs were consistent with those expected from assemblies of Si nanocrystals with a size dispersion and different surface passivation.

## Competing interests

The authors declare that they have no competing interests.

## Authors’ contributions

IL is a Ph.D. student who made the experiments and wrote a first draft of the manuscript. AO performed PL measurements, while AGN supervised the work and corrected, completed, and fully edited the paper. All authors read and approved the final manuscript.
